# APOE ε4 Modifies Effect of Residential Greenness on Cognitive Function among Older Adults: A Longitudinal Analysis in China

**DOI:** 10.1038/s41598-019-57082-7

**Published:** 2020-01-09

**Authors:** Anna Zhu, Lijing Yan, Chang Shu, Yi Zeng, John S. Ji

**Affiliations:** 1 Environmental Research Center, Duke Kunshan University, Kunshan, China; 2 Global Health Research Center, Duke Kunshan University, Kunshan, China; 3 School of Medicine, Yale University, New Haven, Connecticut USA; 4 Center for the Study of Aging and Human Development, Duke Medical School, Durham, NC USA; 5 Center for Healthy Aging and Development Studies, National School of Development, Peking University, Beijing, China; 6 Nicholas School of the Environment, Duke University, Durham, NC USA

**Keywords:** Neurological disorders, Epidemiology

## Abstract

We tested whether the protective effects of residential greenness on cognitive function differ by APOE ε4 status by using the Chinese Longitudinal Healthy Longevity Survey (CLHLS). We calculated Normalized Difference Vegetation Index (NDVI) using 500 m radii around residential addresses to measure greenness, and the Mini-Mental State Examination (MMSE) to assess cognitive function. We dichotomized the participants into APOE non-ε4 carriers, and APOE ε4 carriers. We applied the generalized estimating equations (GEE) to examine the association between baseline annual average NDVI, APOE ε4 carrier status, and cognitive impairment. Among 6,994 participants, 19.30% were APOE ε4 carriers. Compared to APOE ε4 non-carriers, the APOE ε4 carriers had a 15% higher odds of cognitive impairment (OR: 1.15, 95% CI: 1.05, 1.26). There was an age difference; the protective effect of residential greenness (the highest vs. lowest quartile) on cognitive impairment was observed among the non-ε4 carriers (OR: 0.83, 95% CI: 0.72, 0.95), but not among the ε4 carriers (OR: 1.00, 95% CI: 0.74, 1.34). However, the interaction term between annual average NDVI and APOE ε4 status was not significant (OR: 1.04, 95% CI: 0.97, 1.11). The protective effects of residential greenness on cognitive function differed by APOE ε4 status, which elucidated possible gene-environment interaction mechanisms in which residential greenness may benefit health.

## Introduction

Natural environment may benefit cognitive function by reducing stress and restoring attention^1^. There is evolving evidence on the association between higher levels of residential greenness and better cognitive function, although the evidence was not consistent. Two studies of 884 adults aged 65 years and older in the United States (US)^2^, and 1091 adults aged 70 years and older in Scotland illustrated the protective associations^3^. But another two studies of 949 adults aged 50 years and older in Chicago, US^4^, and 6,658 adults aged 40 to 69 years in Quebec, Canada found no association^5^. The inconsistent findings probably were due to their different assessments of residential greenness and cognitive function.

Apolipoprotein E (APOE) gene polymorphism is a well-known genetic risk factor for cognitive decline^6^. APOE is a polymorphic apolipoprotein essential for plasma lipoprotein metabolism and lipid transport. Defined by the single nucleotide polymorphisms (SNPs) of rs429358 and rs7412, there are three common allelic variants of APOE, including APOE ε2, APOE ε3, and APOE ε4^7^. APOE gene allele distribution varies among different ethnic groups. APOE ε4 is more frequent among people residing in Northern Europe than in Mediterranean regions and Asia. APOE ε3 is the most frequent among all groups, while APOE ε2 is more common among Africans^7,8^. With regards to health effects, carriage of APOE ε4 allele is highly predictive of cognitive decline, including Alzheimer’s disease (AD)^9^. The association has been supported by evidence of neuroimaging markers. A systematic review and meta-analysis of 14 cross-sectional studies between 1996 and 2014 found that APOE ε4 carriers had atrophic hippocampal volume, increased cerebral amyloid deposition, and cerebral hypometabolism^10^.

Most diseases are thought to be caused by environmental risks, genetic risks, and their interaction^11^. It has been hypothesized that genes explain 50% to 70% of the variation in cognitive function but their heritability differs by the environment^12^. A number of studies have reported the effect modification of APOE ε4 status on the association between environmental risks and cognitive function. These studies mainly explored lifestyle factors like diet^13^, physical activity^14^, and socioeconomic contexts^15,16^. However, the interaction between APOE ε4 status and the built environment, including residential greenness, has not been reported although residential greenness benefits population health. In addition, gene-environment interactions are essential for the progression of cognitive impairment. Studying the interaction between APOE and residential greenness would help explore the biological mechanisms, and better understand the estimates of their health effects on cognitive function. Our study aimed to assess whether the association between residential greenness and cognitive function differ by APOE ε4 status among Chinese older adults aged 65 years and older.

## Methods

### Study population

We used the survey and genetic data (participant study entry between 2000 and 2014) from the Chinese Longitudinal Healthy Longevity Survey (CLHLS). The CLHLS is a prospective longitudinal cohort created to explore determinants of healthy longevity in Chinese older adults. The CLHLS used a multistage, stratified cluster sampling, and recruited participants from 22 out of 31 provinces in China. With 631 cities and counties randomly selected as the sample sites, the study sample represents about 85% of the Chinese population. The survey only included the older adults aged 80 years and older in 1998 and 2000, and expanded to the older adults aged 65 years and older since the 2002 survey. The CLHLS oversampled the older adults aged 80 years and older. A more detailed description of the sampling design could be found elsewhere^17^. Initiated in 1998, the CLHLS conducted followed-up surveys among the survivals and recruited new participants in 2000, 2002, 2005, 2008, 2011, 2014, and 2018. CLHLS has collected extensive data, including demographic characteristics, socioeconomic status, lifestyle, physical health, psychological well-being, survival status, biomarkers, and gene^17^.

There were 19,726 older adults aged 65 years and older with follow-up surveys and detailed information on residential greenness. We excluded the participants who were without genetic data (n = 12,640), and were missing the SNPs of rs7412 (n = 92). We included 6,994 participants for the analysis of residential greenness, APOE ε4 status, and cognitive function.

### Greenness assessment

We calculated the Normalized Difference Vegetation Index (NDVI) to reflect residential greenness. The plants absorb visible light and leaves reflect near-infrared light in the process of photosynthesis. NDVI is the ratio of the difference between the near-infrared region and red visible reflectance to the sum of these two measures. NDVI ranges from −1.0 to 1.0, with larger values indicating higher levels of vegetative density^18^. Different NDVI values indicate different environments. Negative values often refer to blue space or water; values of 0.1 and below reflect barren areas of rock, sand, or snow; values of 0.2 to 0.4 represent shrub and grassland; while higher values indicate temperate and tropical rainforests^19^.

We obtained NDVI values from the Moderate-Resolution Imaging Spectro-Radiometer (MODIS) in the National Aeronautics and Space Administration’s Terra Satellite. MODIS has a temporal resolution of 16 days. We calculated two NDVI values for January, April, July, and October between 2000 and 2014 to reflect seasonal variation in greenness. We calculated the NDVI values in the 500-m radius around the residence. We calculated the baseline annual average NDVI to indicate residential greenness at baseline. In addition, we calculated the quartiles of NDVI and 0.1-unit of NDVI values for the statistical analysis.

### Cognitive function assessment

Our health outcome was cognitive function, assessed by the adapted Chinese version of Mini-Mental State Examination (MMSE). MMSE evaluated orientation, registration, attention and calculation, recall, and language^20^. The reliability and validity of MMSE have been demonstrated by previous studies^21,22^. There were 24 self-reported questions in our MMSE (Supplementary Table S1). Each question scored as zero (wrong or unable to answer) or one (correct). We converted the 24-item MMSE to a scale from 0 to 30 for consistency with other studies^23,24^. We categorized cognitive function into groups of having normal cognition (MMSE scores > = 24, as the reference group), and cognitive impairment (MMSE <24)^25^. MMSE was repeatedly measured in 2000, 2002, 2005, 2008, 2011, and 2014 during the follow-up period. We reported baseline MMSE (cognitive function at study entry) and final MMSE (cognitive function at the last available survey).

### APOE ε4 status

CLHLS collected DNA samples from parts of participants in 1998, 2000, 2002, 2005, 2008–2009, and 2011–2012. Genotyping of DNA samples was produced by Beijing Genomics Institute (BGI). APOE SNPs rs429358 and rs7412 were genotyped by the Illumina HumanOmniZhongHua-8 BeadChips. This chip could profile over 900 K SNPs per sample. 98.9% of SNPs were international compatible. In the process of sample filtering, the samples whose call rate was less than 95%, identity-by-state probabilities with PI_HAT was > 0.25, and minor allele frequency was less than 1% were excluded. In addition, principal components analysis was used to test whether there was an ancestry difference among the sample participants. More details on the genotyping platform, sample filtering, and quality control can be found in Zeng *et al*.^26^. Determined by rs429358 and rs7412, there are six APOE genotypes in our participants, including ε2/ε2, ε2/ε3, ε2/ε4, ε3/ε3, ε3/ε4, and ε4/ε4. We dichotomized the participants into APOE non-ε4 carriers (including ε2/ε2, ε2/ε3, and ε3/ε3), and ε4 carriers (including ε2/ε4, ε3/ε4 and ε4/ε4).

### Covariates

We assessed a number of baseline characteristics including age, gender, marital status, urban/rural residence, education, occupation, financial support, social and leisure activity, smoking status, alcohol consumption, and physical activity. We measured age based on the difference between the interview dates, and the verified birth dates. We dichotomized marital status to married and not married at the time of the interview (separated, or divorced, or widowed, or never married). We divided residence into urban areas and rural areas. Given the relatively low level of education in our participants, we defined less than one year of schooling as no formal education, and defined one year of schooling or more as some formal education. We categorized occupation into professional work (professional and technical personnel, government, and management), and non-professional work (agriculture, fishing, service, industry, and housework). We generated a binary variable for financial support, including financial independence if the participants were financially independent with their work and retirement wage, and financial dependence if they financially relied on other family members. We calculated social and leisure activity index by taking into consideration seven activities, including gardening, personal outdoor activities excluding exercise, raising poultry or pets, reading, playing cards or *mah-jong*, listening to the radio or watching TV, and participating in organized social activities. Each activity was scored zero (no) or one (yes), and the index ranged from zero to seven^27^. We categorized smoking status into never smokers as those neither smoked in the past nor at the time of the interview, former smokers as those smoked in the past but not at the time of the interview, and current smokers as those smoked at the time of the interview. We assessed alcohol consumption by using a similar definition. We dichotomized physical activity to yes and no.

### Statistical analysis

We used the generalized estimating equations (GEE) to explore whether APOE ε4 carriers were at higher risks of cognitive impairment than APOE non-ε4 carriers. In addition, we also applied GEE to examine the association between baseline annual average NDVI and cognitive function. Stratified analysis by APOE ε4 status (non-ε4 carriers vs. ε4 carriers) was performed. Furthermore, we assessed the effects of annual average NDVI, APOE ε4 status, and their interaction on cognitive function by using GEE. Subgroup analysis by the age group (aged 65 to 79 years vs. aged 80 years and older) was conducted for all the analysis. All the regression models were adjusted for age, gender, marital status, urban/rural residence, education, occupation, financial support, social and leisure activity, smoking status, alcohol consumption, and physical activity, which might bias the association between residential greenness and cognitive function. Cognitive function was repeatedly assessed by using the MMSE during the follow-up period. The participants with normal cognition (MMSE >  = 24) were defined as the reference group.

We calculated the odds ratio (OR), and 95% confidence intervals (CIs) to estimate the magnitude and odds of cognitive impairment, under different levels of residential greenness and APOE ε4 status. We reported the results of quartiles of NDVI and 0.1-unit of NDVI. STATA 14.0 was used for statistical analysis.

### Ethical approvals

The study protocol was approved by the Institutional Review Board, Duke University (Pro00062871), and the Biomedical Ethics Committee, Peking University (IRB00001052-13074). All research was performed in accordance with relevant guidelines and regulations. Paper-based informed consent was signed and collected from all participants.

## Results

### Baseline characteristics of CLHLS participants

In total, there were 6,994 older adults with a mean age of 80 (SD: 10.95) years (Table 1). Our study had slightly more females (51.39%) than males (48.61%). Up to 82.67% were from rural areas. Only 45.41% had one-year or more education, representative of the social context in our participants’ birth years. 80.70% and 19.30% were APOE non-ε4 carriers, and ε4 carriers, respectively. The levels of education did not differ by APOE ε4 status. Mean annual average NDVI at baseline in the 500-m radius around the residence was 0.41 (SD: 0.14). APOE ε4 carriers had similar MMSE scores than the non-ε4 carriers (25.03 vs. 24.97 at baseline, p-value: 0.54). The percentages of current smokers and drinkers were relatively low, which were about 23.28% and 22.88%, respectively.

**Table 1:** Baseline characteristics of CLHLS participants.

Characteristics	Total	APOE non-ε4 carriers	APOE ε4 carriers
N (%)	6,994	5,644 (80.70)	1,350 (19.30)
Baseline annual average NDVI (mean ± SD)	0.41 ± 0.14	0.41 ± 0.14	0.40 ± 0.14
Baseline MMSE scores (mean ± SD)	25.02 ± 6.78	25.03 ± 6.78	24.97 ± 6.77
Final MMSE scores (mean ± SD)	22.12 ± 9.39	22.14 ± 9.40	22.00 ± 9.36
Age (years) (mean ± SD)	80 ± 10.95	80 ± 11.04	79 ± 10.56
Age group			
65–79	3,470 (49.61)	2,760 (48.90)	710 (52.59)
80–89	1,972 (28.20)	1,586 (28.10)	386 (28.59)
90–99	1,093 (15.63)	915 (16.21)	178 (13.19)
> = 100	459 (6.56)	383 (6.79)	76 (5.63)
Gender			
Males	3,400 (48.61)	2,722 (48.23)	678 (50.22)
Females	3,594 (51.39)	2,922 (51.77)	672 (49.78)
Marital status			
Married	3,290 (47.04)	2,629 (46.58)	661 (48.96)
Not married	3,704 (52.96)	3,015 (53.42)	689 (51.04)
Residence			
Urban area	1,212 (17.33)	959 (16.99)	253 (18.74)
Rural area	5,782 (82.67)	4,685 (83.01)	1,097 (81.26)
Occupation			
Professional work	578 (8.26)	469 (8.31)	109 (8.07)
Non-professional work	6,416 (91.74)	5,175 (91.69)	1,241 (91.93)
Education			
Formal education	3,176 (45.41)	2,558 (45.32)	618 (45.78)
No formal education	3,818 (54.59)	3,086 (54.68)	732 (54.22)
Financial support			
Financial independence	2,517 (35.99)	2,010 (35.61)	507 (37.56)
Financial dependence	4,477 (64.01)	3,634 (64.39)	843 (62.44)
Social and leisure activity index (mean ± SD)	2.71 ± 1.49	2.68 ± 1.50	2.84 ± 1.46
Smoking status			
Never smoker	4,442 (63.51)	3,611 (63.98)	831 (61.56)
Former smoker	924 (13.21)	740 (13.11)	184 (13.63)
Current smoker	1,628 (23.28)	1,293 (22.91)	335 (24.81)
Alcohol consumption			
Never drinker	4,762 (68.09)	3,854 (68.28)	908 (67.26)
Former drinker	632 (9.04)	511 (9.05)	121 (8.96)
Current drinker	1,600 (22.88)	1,279 (22.66)	321 (23.78)
Physical activity			
Yes	2,337 (33.41)	1,891 (33.50)	446 (33.04)
No	4,657 (66.59)	3,753 (66.50)	904 (66.96)

### The association between APOE ε4 status and cognitive impairment

Compared to APOE non-ε4 carriers, the ε4 carriers had a 15% higher odds of cognitive impairment (OR: 1.15, 95% CI: 1.05, 1.26) (Table 2). The effects were stronger among the ε4 carriers aged 65 to 79 years (OR: 1.15, 95% CI: 1.00, 1.32) than those aged 80 years and old (OR: 1.13, 95% CI: 1.00, 1.29).

**Table 2:** Association between APOE ε4 status and cognitive impairment.

	n	OR (95% CI)	p-value
APOE ε4 status	6,994		
APOE non-ε4 carriers	5,644	Ref	Ref
APOE ε4 carriers	1,350	1.15 (1.05, 1.26)	0.004
By age group			
Aged 65 to 79 years	3,470		
APOE non-ε4 carriers	2,760	Ref	Ref
APOE ε4 carriers	710	1.15 (1.00, 1.32)	0.048
Aged 80 years and older	3,524		
APOE non-ε4 carriers	2,884	Ref	Ref
APOE ε4 carriers	640	1.13 (1.00, 1.29)	0.054

Note: ORs (95% CIs) of cognitive impairment (MMSE < 24) were shown above.

All the regression models were adjusted for age, gender, marital status, urban/rural residence, education, occupation, financial support, social and leisure activity, smoking status, alcohol consumption, and physical activity at baseline.

### The association between baseline annual average NDVI and cognitive impairment by APOE ε4 status

Compared to the participants living in the lowest quartile of residential greenness, those in the highest quartile had a 15% (95% CI: 0.75, 0.97) lower odds of cognitive impairment (Table 3). The protective effects were only observed among the non-ε4 carriers (OR: 0.83, 95% CI: 0.72, 0.95) but not among the ε4 carriers (OR: 1.00, 95% CI: 0.74, 1.34). In addition, the association between residential greenness and cognitive function also differed by the age group. The significant effects were only observed among the older adults aged 65 to 79 years (OR of the highest quartile of NDVI: 0.76, 95% CI: 0.62, 0.93) but not those aged 80 years and older (OR of the highest quartile of NDVI: 0.94, 95% CI: 0.80, 1.10). Furthermore, the similar associations were also found for each 0.1 unit increase in baseline annual average NDVI.

**Table 3:** Association between baseline annual average NDVI and cognitive impairment by APOE ε4 status.

Exposure metrics	All participants		APOE non-ε4 carriers		APOE ε4 carriers	
All participants	n	OR (95% CI)	n	OR (95% CI)	n	OR (95% CI)
Quartiles of NDVI	6,994		5,644		1,350	
Quartile 1 (−0.06, 0.32)	1,750	Ref	1,385	Ref	365	Ref
Quartile 2 (0.32, 0.44)	1,747	1.14 (1.01, 1.28)	1,407	1.12 (0.99, 1.28)	340	1.22 (0.94, 1.59)
Quartile 3 (0.44, 0.52)	1,750	1.03 (0.91, 1.16)	1,421	0.97 (0.85, 1.11)	329	1.29 (0.97, 1.72)
Quartile 4 (0.52, 0.76)	1,747	0.85 (0.75, 0.97)	1,431	0.83 (0.72, 0.95)	316	1.00 (0.74, 1.34)
0.1 unit of NDVI	/	0.96 (0.93, 0.99)	/	0.95 (0.92, 0.98)	/	1.02 (0.94, 1.10)
By age group						
Aged 65 to 79 years						
Quartiles of NDVI	3,470		2,760		710	
Quartile 1 (−0.06, 0.32)	960	Ref	748	Ref	212	Ref
Quartile 2 (0.32, 0.44)	910	1.17 (0.98, 1.39)	724	1.17 (0.96, 1.42)	186	1.15 (0.80, 1.66)
Quartile 3 (0.44, 0.52)	844	0.97 (0.80, 1.16)	677	0.91 (0.74, 1.11)	167	1.20 (0.80, 1.81)
Quartile 4 (0.52, 0.76)	756	0.76 (0.62, 0.93)	611	0.72 (0.57, 0.90)	145	0.92 (0.60, 1.41)
0.1 unit of NDVI	/	0.94 (0.89, 0.98)	/	0.92 (0.87, 0.97)	/	0.99 (0.89, 1.11)
Aged 80 years and older						
Quartiles of NDVI	3,524		2,884		640	
Quartile 1 (−0.06, 0.32)	790	Ref	637	Ref	153	Ref
Quartile 2 (0.32, 0.44)	837	1.10 (0.95, 1.28)	683	1.08 (0.91, 1.28)	154	1.27 (0.90, 1.79)
Quartile 3 (0.44, 0.52)	906	1.07 (0.91, 1.25)	744	1.02 (0.86, 1.22)	162	1.40 (0.97, 2.03)
Quartile 4 (0.52, 0.76)	991	0.94 (0.80, 1.10)	820	0.90 (0.76, 1.08)	171	1.12 (0.76, 1.64)
0.1 unit of NDVI	/	0.98 (0.94, 1.02)	/	0.97 (0.93, 1.01)	/	1.05 (0.95, 1.17)

Note: ORs (95% CIs) of cognitive impairment (MMSE <24) were shown above.

All the regression models were adjusted for age, gender, marital status, urban/rural residence, education, occupation, financial support, social and leisure activity, smoking status, alcohol consumption, and physical activity at baseline.

### The interaction between baseline annual average NDVI and APOE ε4 status on cognitive impairment

Model 1 showed that the risk of cognitive impairment of APOE ε4 carriers vs. non-ε4 carriers (OR: 1.15, 95% CI: 1.05, 1.26). Since one year increase in age has a 6% increased odds of cognitive impairment (OR: 1.06, 95% CI: 1.06, 1.07) in our model, the effect of APOE ε4 carriers was equivalent to 2.5 additional years of aging. At the same time, each 0.1-unit increase in NDVI has a 4% decreased odds of cognitive impairment (OR: 0.96, 95% CI: 0.93. 0.99), and thus the effect of APOE ε4 carriers was equivalent to decreasing greenness by 0.38 units (Table 4). However, the interaction term between each 0.1 unit increase in baseline annual average NDVI and APOE ε4 status did not achieve statistical significance (OR: 1.04, 95% CI: 0.97, 1.11, p-value for interaction: 0.281).

**Table 4:** The interaction between baseline annual average NDVI and APOE ε4 status on cognitive impairment (n = 6,994).

	Model 1*		Model 2**	
	OR (95% CI)	p-value	OR (95% CI)	p-value
0.1 unit of NDVI	0.96 (0.93, 0.99)	0.017	0.95 (0.92, 0.99)	0.008
APOE ε4 status				
APOE non-ε4 carriers	Ref	/	Ref	/
APOE ε4 carriers	1.15 (1.05, 1.26)	0.004	0.99 (0.74, 1.32)	0.927
Interaction term	/	/		
APOE non-ε4 carriers *0.1 unit of NDVI			Ref	/
APOE ε4 carriers *0.1 unit of NDVI			1.04 (0.97, 1.11)	0.281
By age group				
Aged 65 to 79 years (n = 3,470)				
0.1 unit of NDVI	0.94 (0.89, 0.98)	0.009	0.92 (0.88, 0.97)	0.004
APOE ε4 status				
APOE non-ε4 carriers	Ref	/	Ref	/
APOE ε4 carriers	1.15 (1.00, 1.32)	0.054	0.89 (0.59, 1.34)	0.588
Interaction term	/	/		
APOE non-ε4 carriers *0.1 unit of NDVI			Ref	/
APOE ε4 carriers *0.1 unit of NDVI			1.06 (0.96, 1.17)	0.213
Aged 80 years and older (n = 3,524)				
0.1 unit of NDVI	0.98 (0.94, 1.03)	0.425	0.98 (0.94, 1.02)	0.368
APOE ε4 status				
APOE non-ε4 carriers	Ref	/	Ref	/
APOE ε4 carriers	1.13 (1.00, 1.29)	0.055	1.05 (0.69, 1.57)	0.831
Interaction term	/	/		
APOE non-ε4 carriers *0.1 unit of NDVI			Ref	/
APOE ε4 carriers *0.1 unit of NDVI			1.02 (0.93, 1.12)	0.685

Note: ORs (95% CIs) of cognitive impairment (MMSE < 24) were shown above.

*Model 1 examined the effects of baseline annual average NDVI and APOE ε4 status;

**Model 2 examined the effects of baseline annual average NDVI, APOE ε4 status, and their interaction.

All the regression models were adjusted for age, gender, marital status, urban/rural residence, education, occupation, financial support, social and leisure activity, smoking status, alcohol consumption, and physical activity at baseline.

## Discussion

Our study found the APOE ε4 carriers had a higher risk of cognitive impairment, and did not benefit from the protective effects of residential greenness, compared with the non-ε4 carriers. In addition, the risk of cognitive impairment of APOE ε4 carriers vs. non-ε4 carriers was equivalent to 2.5 additional years of aging, and also equivalent to decreasing greenness by 0.38 units. Our findings reinforced evidence on the higher risks of APOE ε4 allele carriage on cognitive impairment among older adults.

The association between higher levels of residential greenness and lower risks of cognitive impairment was significant only among the APOE non-ε4 carriers but not the ε4 carriers. Our finding suggests that the detrimental effects of APOE ε4 allele carriage might overwhelm the protective effects of residential greenness. The different risk patterns between the environmental risks and cognitive function by APOE ε4 status were consistent with other studies. The Cardiovascular Health Cognition Study (1992–2000) in the US, composed of 3,375 adults aged 65 years and older, reported the protective effects of physical activity on dementia only among the APOE non-ε4 carriers but not the ε4 carriers^14^. The Women’s Health Initiative Memory Study, a US-wide cohort of older women, showed that there were stronger adverse effects of PM2.5 on the cognitive decline and all-cause dementia among the APOE ε4/ ε4 carriers^28^. The Cardiovascular Health Study, involving of 3,393 Medicare-eligible women aged 65 years and older, found that estrogen use was related to less cognitive decline only among the APOE non-ε4 carriers, rather than the ε4 carriers^29^.

The biological mechanisms of the association between residential greenness and cognitive function modified by APOE ε4 status were unclear due to the limited evidence. Residential greenness and APOE ε4 allele carriage may have offsetting effects on cognitive function. There was evidence of an interaction between atherosclerosis and APOE ε4 status on AD^30^. The detrimental effects of atherosclerosis on AD were stronger among the APOE ε4 carriers. APOE ε4 allele carriage was related to higher risks of atherosclerosis. However, residential greenness was independently related to lower levels of sympathetic activation, reduced oxidative stress, and higher angiogenic capacity, which ultimately benefited atherosclerosis^31^. Additionally, residential greenness might affect cognitive function through the promoted physical activity^32^, and reduced environmental pollution^33^. Physical activity was associated with specific gene expression for promoting neurogenesis and neural plasticity^34^. Furthermore, more green space could reduce air pollution, like particular matters, which had adverse effects on cognitive function. While APOE ε4 allele carriage was associated with increased susceptibility to air pollutants, which might offset the protective effects of residential greenness^28^. Our study lacked biological evidence. Further experiment research is warranted.

There was an age difference in the effects of residential greenness and APOE ε4 status on cognitive function. The protective effects of residential greenness on cognitive function were only observed among the adults aged 65 to 79 years. Cognitive function declined over age. Adults aged 80 years and older had more prevalent and severe cognitive impairment^35^. The association between residential greenness and cognitive function was relatively weak. It was likely that the protective effects of residential greenness on more severe cognitive impairment may be negligible. In addition, the risks of APOE ε4 allele carriage on cognitive impairment were only significant among the adults aged 65 to 79 years but not for the adults aged 80 years and older, although the association was weak. This was consistent with other studies^36,37^. A study of 553 adults aged 85 years and older in Finland, reported no association between APOE ε4 allele carriage and development of dementia, or cognitive decline among the initially non-demented persons or the whole group of survivors. But there were greater cognitive declines in APOE ε4 carriers than non- ε4 carriers among the demented participants. The authors argued that no association might be due to the robustness of the MMSE test. It was also possible that the effect of APOE ε4 allele carriage on cognitive functions was age-dependent and worn out when at advanced old age^37^.

Our participants are highly unique compared to other cohorts in developed countries, as our study participants had an extremely low level of educational attainment. Only 45.45% had one-year or more formal education. The mean age of our study participants was 80 years old at baseline, meaning that they were born in early 20^th^ century, during a time of warfare and disrupted social structures. During this turbulent time, society had little capacity to provide primary school education. In fact, documented primary school enrollment ratio increased from 1.9% in 1907 to 9.4% in 1916 to 22.6% in 1933^38^. While our coding of educational attainment may appear crude, it does reflect the special contexts of study participants’ experiences.

Our study has several limitations. First, although NDVI could objectively reflect the density of vegetation, it could not indicate specific types of vegetation. We were not sure how different types of vegetation could influence the association. We used the NDVI at 500 m radius around the residential address to indicate residential greenness. We were also not sure whether other scales affected the association. Second, MMSE may be insensitive to mild cognitive impairment. The validation of MMSE was influenced by the different versions, administration, scoring, and interpretation. Restricted by data availability in the CLHLS, we could not verify the cognitive function by the clinical diagnosis^21,22^. However, it has been demonstrated that the adapted Chinese version used in our study was reliable and valid, and has been used to assess the cognitive function in a number of health studies^24,39^. Third, there might be selection bias. Only parts of our participants, mainly from the longevity areas, had genetic data. They may be healthier, like having a better cognitive function. Additionally, since our study used a longitudinal analysis, we only included the participants with at least one follow-up survey, who were younger and had a better cognitive function at baseline, than those who died or were lost after the baseline survey. The percentage of current smokers and drinkers were relatively low, about 23.28% and 22.88%, respectively. Since smoking and alcohol consumption are high risks of health, there might be health selection effects.

There are several strengths to our study. To our knowledge, this is the first large-scale population-based cohort study to explore the interaction between residential greenness and APOE ε4 status on cognitive function among older adults. Our study reinforced the evidence of higher risks of APOE ε4 allele carriage on cognitive impairment, and provided extra evidence on gene-environment interaction. In addition, we used a longitudinal design, with multiple measurements of cognitive function and a long follow-up period from 2000 to 2014. Furthermore, SES is unlikely to bias our analysis. One unique context in our study is that the participants living in greener areas tended to have lower SES, like lower levels of education, which were associated with better cognitive function. APOE ε4 status did not differ by SES. Therefore, the associations between residential greenness, APOE ε4 status, and cognitive function is unlikely to be explained by SES.

## Conclusions

Our study found that the protective effects of residential greenness on cognitive function among older adults differed by APOE ε4 status, with the significant association only observed among the APOE non-ε4 carriers. Our findings suggest that APOE ε4 allele carriage has greater effects on cognitive impairment than residential greenness, which was observed only among the older adults aged 65 to 79 years old. Our study may help elucidate possible gene-environment interaction mechanisms in which residential greenness may benefit health.

## Supplementary information

Supplementary information.

## Data Availability

The CLHLS data can be obtained by reasonable request on request from the Center for Healthy Aging and Development Studies, Peking University.
