# Supplementary material for: APOE ε4 Modifies Effect of Residential Greenness on Cognitive Function among Older Adults: A Longitudinal Analysis in China
Source: Sci Rep. 2020 Jan 9;10:82. doi: 10.1038/s41598-019-57082-7 (PMC6952401; doi:10.1038/s41598-019-57082-7)
Supplement: Supplementary file 1 — Supplementary information. [file 41598_2019_57082_MOESM1_ESM.docx]

**Supplementary Information**

**Title: APOE ε4 Modifies Effect of Residential Greenness on Cognitive Function among Older Adults: A Longitudinal Analysis in China**

Anna Zhu, Lijing Yan, Chang Shu, Yi Zeng, John S. Ji*

**Supplementary Table S1 The list of the 24 MMSE questions in the CLHLS**

| Questions | Point |
| --- | --- |
| What is the time of day right now (morning, noon, afternoon, or evening)? | 1 point |
| What is the month right now? | 1 point |
| What is the date of the mid-autumn festival? | 1 point |
| What is the season right now? | 1 point |
| What is the name of this county or district? | 1 point |
| Number of food listed in one minute | 1 point |
| Repeat the name of "table", "apple", and "clothes" | 3 points |
| Count down from twenty by threes, 1 point for each correct. Stop after 5 answers | 5 points |
| Draw the figure following the sample | 1 point |
| Recall the name of "table", "apple", and "clothes" | 3 points |
| Name a pencil, and a watch | 2 points |
| Repeat the sentence of “You harvest what you plant” | 1 point |
| Follow a 3-stage command: “Take a paper in your right hand, fold it in half, and put it on the floor” | 3 points |

**Supplementary Table S2 The subgroup analysis of baseline annual average NDVI, APOE ε4 status, and cognitive impairment**

|  | All participants | | APOE non-ε4 carriers | | APOE ε4 carriers | |
| --- | --- | --- | --- | --- | --- | --- |
| All participants | n | OR (95% CI) | n | OR (95% CI) | n | OR (95% CI) |
| Quartiles of NDVI | 6,994 |  | 5,644 |  | 1,350 |  |
| Quartile 1 | 1,750 | Ref | 1,385 | Ref | 365 | Ref |
| Quartile 2 | 1,747 | 1.14 (1.01, 1.28) | 1,407 | 1.12 (0.99, 1.28) | 340 | 1.22 (0.94, 1.59) |
| Quartile 3 | 1,750 | 1.03 (0.91, 1.16) | 1,421 | 0.97 (0.85, 1.11) | 329 | 1.29 (0.97, 1.72) |
| Quartile 4 | 1,747 | 0.85 (0.75, 0.97) | 1,431 | 0.83 (0.72, 0.95) | 316 | 1.00 (0.74, 1.34) |
| 0.1 unit of NDVI | / | 0.96 (0.93, 0.99) | / | 0.95 (0.92, 0.98) | / | 1.02 (0.94, 1.10) |
|  |  |  |  |  |  |  |
| By occupation |  |  |  |  |  |  |
| Professional work | | | | | | |
| Quartiles of NDVI | 578 |  | 469 |  | 109 |  |
| Quartile 1 | 294 | Ref | 234 | Ref | 60 | Ref |
| Quartile 2 | 122 | 0.97 (0.61, 1.53) | 101 | 0.97 (0.59, 1.62) | 21 | 0.95 (0.28, 3.24) |
| Quartile 3 | 76 | 1.28 (0.69, 2.39) | 65 | 0.96 (0.55, 1.68) | 11 | 2.00 (0.32, 12.63) |
| Quartile 4 | 86 | 1.41 (0.82, 2.45) | 69 | 1.21 (0.67, 2.19) | 17 | 2.48 (0.69, 8.93) |
| 0.1 unit of NDVI | / | 1.07 (0.93, 1.24) | / | 0.99 (0.85, 1.15) | / | 1.40 (1.03, 1.91) |
|  |  |  |  |  |  |  |
| Non-professional work | | | | | | |
| Quartiles of NDVI | 6,416 |  | 5,175 |  | 1,241 |  |
| Quartile 1 | 1,456 | Ref | 1,151 | Ref | 305 | Ref |
| Quartile 2 | 1,625 | 1.14 (1.01, 1.29) | 1,306 | 1.13 (0.98, 1.29) | 319 | 1.23 (0.94, 1.61) |
| Quartile 3 | 1,674 | 1.02 (0.90, 1.15) | 1,356 | 0.97 (0.85, 1.11) | 318 | 1.26 (0.95, 1.67) |
| Quartile 4 | 1,661 | 0.84 (0.74, 0.96) | 1,362 | 0.82 (0.71, 0.94) | 299 | 0.97 (0.72, 1.31) |
| 0.1 unit of NDVI | / | 0.96 (0.93, 0.99) | / | 0.95 (0.91, 0.98) | / | 1.01 (0.93, 1.08) |
|  |  |  |  |  |  |  |
| By smoking status |  |  |  |  |  |  |
| Never smoker |  |  |  |  |  |  |
| Quartiles of NDVI | 4,442 |  | 3,611 |  | 831 |  |
| Quartile 1 | 1,103 | Ref | 867 | Ref | 236 | Ref |
| Quartile 2 | 1,072 | 1.19 (1.03, 1.36) | 865 | 1.18 (1.01, 1.38) | 207 | 1.26 (0.92, 1.72) |
| Quartile 3 | 1,124 | 0.99 (0.86, 1.14) | 924 | 0.92 (0.78, 1.08) | 200 | 1.42 (1.02, 1.99) |
| Quartile 4 | 1,143 | 0.85 (0.73, 0.99) | 955 | 0.81 (0.69, 0.96) | 188 | 1.07 (0.74, 1.55) |
| 0.1 unit of NDVI | / | 0.95 (0.92, 0.99) | / | 0.93 (0.89, 0.97) | / | 1.06 (0.96, 1.17) |
|  |  |  |  |  |  |  |
| Former & current smokers | | | | | | |
| Quartiles of NDVI | 2,552 |  | 2,033 |  | 519 |  |
| Quartile 1 | 647 | Ref | 518 | Ref | 129 | Ref |
| Quartile 2 | 675 | 1.06 (0.86, 1.30) | 542 | 1.03 (0.82, 1.30) | 133 | 1.22 (0.78, 1.92) |
| Quartile 3 | 626 | 1.09 (0.88, 1.35) | 497 | 1.10 (0.87, 1.39) | 129 | 1.06 (0.64, 1.75) |
| Quartile 4 | 604 | 0.86 (0.69, 1.07) | 476 | 0.85 (0.66, 1.10) | 128 | 0.86 (0.53, 1.40) |
| 0.1 unit of NDVI | / | 0.98 (0.92, 1.03) | / | 0.99 (0.92, 1.05) | / | 0.93 (0.82, 1.05) |
